# Low-cost electronic DC load module design for battery capacity evaluation

**DOI:** 10.1016/j.ohx.2025.e00679

**Published:** 2025-07-17

**Authors:** Minh Nhat Huynh, Quoc Minh Lam, Cong Toai Truong, Huy Hung Nguyen, Van Tu Duong

**Affiliations:** a Key Laboratory of Digital Control and System Engineering (DCSELab), Faculty of Mechanical Engineering, Ho Chi Minh City University of Technology (HCMUT), Vietnam; b Vietnam National University Ho Chi Minh City, Linh Trung Ward, Thu Duc District, Ho Chi Minh City, Vietnam; c Faculty of Electronics and Telecommunication, Saigon University, Ho Chi Minh City, Vietnam

**Keywords:** Low-cost, electronic DC Load, Battery capacity evaluation, Modularized management system, Power management

## Abstract

Rapid advancements in energy storage technology spurred by the use of electricity in a variety of applications have brought attention to the critical need for precise battery capacity evaluation. The electronic DC load devices play an important role in those tests by replicating real-world discharge conditions. However, commercial DC load systems are often prohibitively expensive and remain largely inaccessible to small enterprises, academic laboratories, and independent researchers. While open-source alternatives offer cost advantages, many existing designs lack scalability, flexibility, and ease of use. This study proposes a low-cost, modular electronic DC load capable of continuous operation at up to 50W per module. With its user-friendly interface and support for numerous other tests, including constant current, constant resistor, constant power, battery evaluation, and high-power pulse charge (HPPC) the proposed electronic DC load is robust and simple to use for battery research and evaluation.

## Graphical abstract

**Figure:**
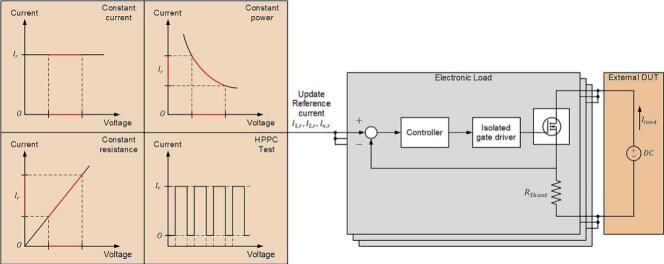

Specifications table

**Table:** 

Hardware name	Master-slave electronic load
Subject area	• Educational tools and open source alternatives to existing infrastructure • General
Hardware type	• Electrical engineering and computer science
Closest commercial analog	Resistor Load, Electronic Load Testing device
Open source license	CC BY 4.0
Cost of hardware	*$282.108*
Source file repository	https://doi.org/10.17632/p2t6vvyvzv.2

## 1 Hardware in context

Battery capacity evaluation is a fundamental process in energy storage research and applications [1]. This evaluation, often performed through discharge or load tests, determines the actual capacity of a battery compared to its nominal value by discharging it under controlled conditions [2]. Various testing techniques have been developed to measure battery performance, including constant current (CC) discharge, constant power (CP) discharge, and constant resistance (CR) discharge, each suited for different application requirements [3,4]. Additionally, hybrid pulse power characterization (HPPC) tests provide insights into a battery’s internal resistance and dynamic response, making them essential for high-power applications such as electric vehicles and renewable energy storage systems [5]. The growing demand for reliable battery performance in consumer electronics, electric mobility, and grid storage has intensified the need for precise and efficient battery testing solutions.

To conduct these evaluations, electronic DC loads have become indispensable tools. These devices can simulate different discharge conditions by maintaining constant current, power, or resistance, ensuring accurate battery characterization. In addition, electronic DC loads facilitate high power pulse charge (HPPC) testing, wherein pulsed currents are applied to assess real-time battery performance across various states of charge. The adaptability of electronic DC loads makes them suitable for both laboratory research and industrial applications, facilitating battery optimization for higher efficiency, extended lifespan, and improved reliability [6].

Despite their advantages, commercial electronic DC loads present notable limitations. The primary concern is cost, as high-end models from Keysight, Chroma, and ITECH are priced between $1000 and $10,000, making them inaccessible to small-scale researchers, independent developers, and budget-constrained organizations [[7], [8], [9]]. Furthermore, these testing devices are closed-source, meaning their firmware and hardware cannot be modified to accommodate specialized research requirements [10]. This lack of flexibility restricts their use in advanced battery studies, where researchers may need customized cycling tests, adaptive load profiles, or integration with external monitoring systems.

To address these limitations, this study proposes an open-source, low-cost electronic DC load that provides a flexible and scalable alternative to commercial solutions. The testing device features a modular architecture, with each module capable of handling up to 50W of power, and multiple units can be interconnected for higher power requirements. In addition, each module supports up to 20A of current and operates within a voltage range of 1V to 50V. The minimum achievable load resistance is limited by the most restrictive condition among the device under test (DUT) supply voltage, the available current, and the maximum power rating. Unlike commercial solutions, it is fully customizable, allowing users to modify both hardware and software for specific testing needs. A key feature of the testing device is the integration of an STM microcontroller, which offers a straightforward programming environment supported by extensive documentation and an active development community. This ensures that even users with limited experience in embedded systems can easily program and adapt the testing device to different testing scenarios.

The technical novelty of this design lies in its cost-effectiveness, modular architecture, and open-source framework. Unlike traditional commercial devices, which have fixed power capacities and proprietary control interfaces, this testing device allows users to scale power handling as needed, customize firmware for specialized test protocols, and integrate additional features such as remote monitoring and data logging. The open-source nature of the project makes it particularly attractive for research institutions, energy startups, and independent hardware developers, offering a level of flexibility that is currently unavailable in off-the-shelf commercial products.

By providing a low-cost, scalable, and fully customizable electronic DC load, this testing device serves as a practical alternative to expensive commercial solutions, meeting the growing demand for flexible and affordable battery testing tools. Its adaptability and accessibility enable broader adoption in experimental research and industrial applications, ensuring that high-quality battery testing is no longer limited to well-funded laboratories but can be widely utilized by engineers, researchers, and developers across various fields.

## 2 Hardware description

### 2.1 System overview

The master–slave electronic load (MSEL) is designed to address the limitations of existing electronic loads, such as high cost, lack of scalability, and limited support for battery testing applications. The key components of MSEL are slave modules, which are designed specifically to handle direct current using power MOSFETs. The module regulates the loading current by adjusting the conductivity of its MOSFETs based on real-time feedback. To enable scalability, slave modules are capable of operating in parallel under the supervision of a master unit, increasing the device’s power-handle capability. The master unit is responsible for monitoring the entire system performance, distributing current between all slave modules, and transmitting data to external devices via USB connectivity. MSEL provides the expandability by utilizing daisy chain architecture via UART, allowing multiple slave modules to be integrated seamlessly without modifying the hardware structure. This flexibility and expandability make it an ideal solution for small to medium-sized laboratories and enterprises that require cost-effective yet high-performance electronic loads.

For user-friendly operation, MSEL is equipped with a 20 × 4 LCD display and a 4 × 5 keypad, providing direct input control and real-time system monitoring. To ensure stable operation under high-power conditions, an active cooling mechanism with four fans is used to prevent thermal buildup during prolonged operation on power MOSFETs. The entire system is constructed using lightweight acrylic sheets, offering electrical insulation and reduced weight, making it suitable for both research environments and industrial testing applications.

### 2.2 Load circuit description

The slave module is responsible for precise current regulation and real-time system monitoring. To achieve these functions, the module consists of three primary subsystems being the MOSFET control circuit, the power stage, and the measurement systems.

First, the MOSFET control circuit plays a crucial role in ensuring precise, smooth, and real-time current regulation. Instead of relying solely on digital processing, which may introduce latency due to microcontroller limitations, MSEL implements an analog PID controller using op-amps, resistors, and capacitors to adjust the MOSFETs’ conductivity. However, in practical applications, standard op-amp cannot provide appropriate accuracy and fast response to stably control the MOSFETs. To address this, MSEL chooses the AD8630 precision amplifier, a special op-amp, for its high accuracy, low offset voltage, and ability to operate close to the supply rail. This approach ensures that the control signal remains, avoiding digital noise and processing delays that could affect load stability.

Second, the power system consists of four MOSFETs connected in parallel, distributing current to enhance load capacity and reduce thermal stress. However, variations in MOSFET internal parameters lead to uneven current distribution, where some MOSFETs conduct more than others, resulting in localized heating and reduced efficiency. To address this, a passive balancing mechanism is integrated into each module. Each MOSFET is connected with a 0.1Ω shunt resistor, which functions as a current sensor. If a MOSFET draws excessive current, the increased voltage drop across its shunt resistor activates a compensation circuit, reducing $V_{GS}$ to limit conduction. This self-adjusting method ensures even current sharing, preventing overloading of individual MOSFETs without requiring microcontroller intervention.

Regrading real-time monitoring and precise measurement, the proposed testing device continuously samples voltage, current, and temperature data. Voltage measurements are taken directly at the load terminals using a differential amplifier, eliminating potential errors caused by wiring resistance. Current sensing is performed through a low-side shunt resistor, and a 16-bit ADC ensures high-resolution data acquisition for precise load regulation. Temperature monitoring is handled by NTC thermistors, allowing dynamic load adjustments based on thermal conditions, improving system stability and component longevity.

Last, the system incorporates multiple protection mechanisms to enhance safety and prevent damage during fault conditions. At the firmware level, the discharge current and power are actively limited to 20 A and 50 W per module, respectively, ensuring that the load operates within safe boundaries. In battery capacity testing mode, if the user attempts to set a current exceeding the maximum allowable value specified by the DUT, the system prevents the parameter from being saved and prompts the user to enter a valid value within safe limits. In the unlikely event of firmware malfunction or unforeseen electrical faults, each module integrates an independent fuse to interrupt excessive current flow, preventing severe damage to both the electronic load and the DUT. Additionally, power diodes are connected in series with the load terminals to provide reverse polarity protection.

By combining analog PID control, passive current balancing, and high-accuracy measurement, each slave module operates independently yet in synchronization with the master controller, ensuring stable power handling and precision load regulation. The proposed testing device is designed to be scalable, reliable, and adaptable, making it suitable for both power testing and precision measurement applications.

In summary, the proposed hardware offers potential applications such as:

• Low cost and open source, making it suitable for small to medium-sized laboratories and enterprises while offering key features.
• Allowing customization, utilizing daisy chain architecture with UART communication for easy expansion.
• Integrated dynamic current allocation algorithm to balance power dissipation between modules, reducing localized overheating and extending MOSFET lifespan.
• Supports multiple operating modes, including standard load testing and specialized battery testing modes.

## 3 Design files summary

The use of the project source files is summarized in Table 1.

**Table 1:** List of component for the project.

Design file name	File type	Open-source license	Location of the file
MSEL_Mainboard	PDF	CC BY 4.0	https://doi.org/10.17632/p2t6vvyvzv.2
MSEL_Project	ZIP	CC BY 4.0	https://doi.org/10.17632/p2t6vvyvzv.2
Final_assembly	ZIP	CC BY 4.0	https://doi.org/10.17632/p2t6vvyvzv.2
Source_code_master	ZIP	CC BY 4.0	https://doi.org/10.17632/p2t6vvyvzv.2
Source_code_slave	ZIP	CC BY 4.0	https://doi.org/10.17632/p2t6vvyvzv.2
Electrical_BOM	XLSX	CC BY 4.0	https://doi.org/10.17632/p2t6vvyvzv.2

MSEL_Mainboard: This file contains the schematic design of the slave circuit, detailing component specifications, circuit interconnections, power distribution, and control logic, serving as the foundation for hardware design and control implementation.

MSEL_Project: This file includes the SCH and PCB layout for manufacturing, specifying component placement, circuit routing, and layer structure, ensuring signal integrity, power management, and efficient heat dissipation.

Final_assembly: This file contains the 3D model integrating all system components, including PCB, control interface, power supply, cooling fans, and mechanical frame, ensuring precise structural assembly and component compatibility.

Source_code_master: This file contains the firmware for the master microcontroller (MCU), responsible for signal processing, system coordination, and load regulation, ensuring system functionality and performance.

Source_code_slave: This file contains the firmware for the slave MCU, performing load parameter measurement, voltage regulation, and data feedback, operating under commands from the master controller to maintain system stability.

Electrical_BOM: This file contains the Bill of Materials (BOM) for the entire DC electronic load circuit, listing all required electronic components, including part numbers, rated values, quantities, suppliers, and ordering information. It serves as a reference for component procurement and ensures accurate assembly of the tester.

## 4 Bill of materials summary

**Table:** 

Designator	Component	Number	Cost per unit −currency	Total cost − currency	Source of materials	Material type
Frame	Acrylic sheet	*1*	*$8.60*	*$8.60*	*[Custom Supplier]*	Polymer
Cable Gland PG11	Cable Gland PG11	*1*	*$0.27*	*$0.27*	*https://www.amazon.com/AMPELE-Plastic-Waterproof-Adjustable-Gaskets/dp/B08TCD5FPW?th* *= 1*	Polymer
Banana Socket	Banana Plug Socket	*2*	*$1.24*	*$2.48*	*https://www.amazon.com/banana-plug-socket/s?k* *= banana + plug + socket*	Metal
USB 2.0 Connector	USB 2.0 Panel Mount	*1*	*$15.59*	*$15.59*	*https://www.amazon.com/PENGLIN-Connector-Converter-High-Speed-Transfer/dp/B0CQ2CC19G*	Metal
M16 Push Button	M16 Push Button Switch	*2*	*$3.18*	*$6.36*	*https://www.amazon.com/Metal-PushButton-Switch-Panel-Mount/dp/B00SENFEYW*	Metal
4x4 Keypad	4x4 Matrix Keypad	*1*	*$5.99*	*$5.99*	*https://www.amazon.com/DIYables-Membrane-Arduino-ESP8266-Raspberry/dp/B0B376LM5T?utm_source* *=* *chatgpt.com**&th = 1*	Polymer
20x4 LCD	20x4 LCD Display (HD44780)	*1*	*$9.99*	*$9.99*	*https://www.amazon.com/HiLetgo-HD44780-Character-Backlight-InterfaceAdapter/dp/B082Y37WXG*	Semiconductor
Fan Grill	Fan Grill/Cover	*4*	*$7.99/4 items*	*$7.99*	*https://www.amazon.com/PATIKIL-Finger-Grill-Protector-Protective/dp/B0BY2SFWCQ*	Metal
Cooling Fan	Cooling Fan 12 V	*4*	*$9.99*	*$39.96*	*https://www.amazon.com/Powerful-Cooling-DBTB0428B2G-404028* *mm-Bearing/dp/B09DQ4N1Q4*	Composite
M3 Screw for LCD (15 mm)	M3 Screw 15 mm (LCD)	*4*	*$0.08*	*$0.32*	*https://www.amazon.com/uxcell-Plastic-Phillips-Machine-Screws/dp/B012SYAHWG*	Metal
M3 Screw for Fan (25 mm)	M3 Screw 25 mm (Fan)	*16*	*$0.06*	*$0.96*	*https://www.amazon.com/Machine-Screws-25MM-Pack-100/dp/B009TDWN40*	Metal
AC Power Connector	AC Power Socket	*1*	*$6.99*	*$6.99*	*https://www.amazon.com/Screw-Mount-IEC320-Connector-QTEATAK/dp/B07VX6JMYV*	Metal
M3 Screw for AC Connector	M3 Screw 15 mm (AC socket)	*4*	*$0.08*	*$0.32*	*https://www.amazon.com/uxcell-Plastic-Phillips-Machine-Screws/dp/B012SYAHWG*	Metal
Electronic Load Module	Electronic Load Module	*1*	*$109.498*	*$109.498*	*[Custom Supplier]*	Composite
Power Supply Unit	PSU (e.g., 12 V 5A)	*1*	*$12.99*	*$12.99*	*https://www.amazon.com/GALYGG-Switching-Universal-Regulated-Transformer/dp/B06XRBYN4Z/ref* *= sxin_16_pa_sp_search_thematic_sspa?content-id = amzn1.sym.b747a510-73a1-4cf4-a45b-74fc1ab8af95%3Aamzn1.sym.b747a510-73a1-4cf4-a45b-74fc1ab8af95&cv_ct_cx = 12v%2B5a%2Bpower%2Bsupply&keywords = 12v%2B5a%2Bpower%2Bsupply&pd_rd_i = B06XRBYN4Z&pd_rd_r = aa197e02-1f25-4b21-be0b-08c58538e460&pd_rd_w = nv1kA&pd_rd_wg = Jvufh&pf_rd_p = b747a510-73a1-4cf4-a45b-74fc1ab8af95&pf_rd_r = VE5X8VKNCG3RVJ1TB4ZA&qid = 1744252355&sbo = RZvfv%2F%2FHxDF%2BO5021pAnSA%3D%3D&sr = 1*–*2-6024b2a3-78e4-4fed-8fed-e1613be3bcce-spons&sp_csd = d2lkZ2V0TmFtZT1zcF9zZWFyY2hfdGhlbWF0aWM&th = 1*	Metal
MiniF4 STM32F401 Board	STM32F401 Dev Board	*1*	*$15.00*	*$15.00*	*https://www.amazon.com/s?k=STM32F401* *+ Dev + Board*	Semiconductor
STM32 Base Board	STM32 Base Board	*1*	*$10.00*	*$10.00*	*[Custom Supplier]*	Polymer
M3 Brass Standoff (15 mm)	Brass Standoff M3x15mm	*8*	*$0.50*	*$4.00*	*https://www.amazon.com/s?k* *= Brass + Standoff + M3x15mm*	Metal
M3 Nut	M3 Nut	*28*	*$0.03*	*$0.84*	*https://www.amazon.com/M3-nuts/s?k* *= M3 + nuts*	Metal
M4 Screw for PSU (15 mm)	M4 Screw 15 mm	*4*	*$0.10*	*$0.40*	*https://www.amazon.com/Bolt-Base-Tensile-Socket-screws/dp/B00SN32OH8*	Metal
M4 Nut	M4 Nut	*4*	*$0.05*	*$0.20*	*https://www.amazon.com/s?k* *= M4 + nut*	Metal
Crimped Wire	Crimped Wire Cable	*1*	*$0.50*	*$0.50*	*[Custom Supplier]*	Metal/Polymer
AC Power Cable	AC Power Cable	*1*	*$6.99*	*$6.99*	*https://www.amazon.com/Cable-Leader-Universal-IEC320-Listed/dp/B07K8VXG99*	Metal/Polymer
Banana to Alligator Clip	Banana to Alligator Clip	*2*	*$9.99/pair*	*$9.99*	*https://www.amazon.com/Neoteck-Banana-Alligator-Crocodile-Multimeter/dp/B01MRY5VBT*	Metal/Polymer
USB Cable	USB Cable (A to type C)	*1*	*$5.88*	*$5.88*	*https://www.amazon.com/Google-USB-C-Charging-Transfer-Cable/dp/B08964WHH5*	Metal/Polymer
*Total*				*$282.108*		

**Fig. 1:**
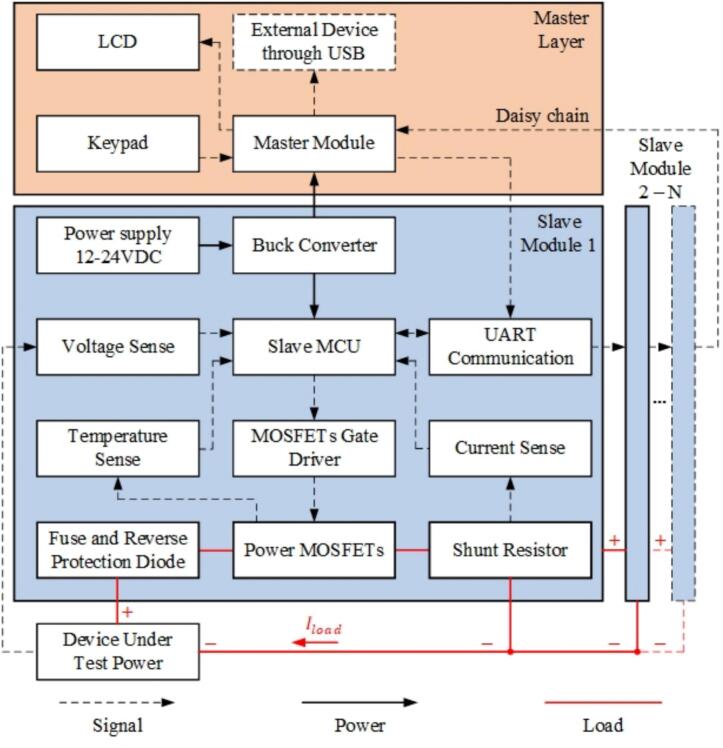
Block diagram of the MSEL hardware architecture.

Bill of electrical materials is provided in the supplementary material, detailing the components required for assembling the electronics module PCB, as exported from the Altium project.Fig. 1

## 5 Build instructions

### 5.1 Installation components

**Fig. 2:**
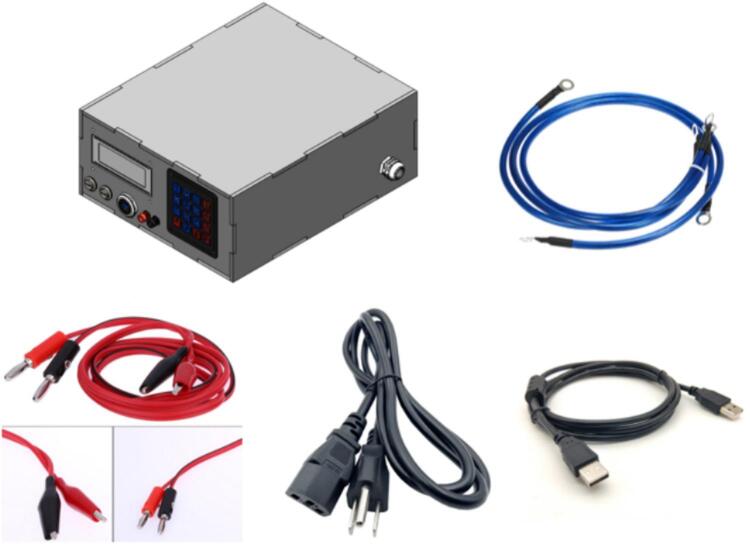
Electronic load testing device and accessories.

The testing device comprises essential components, as illustrated in Fig. 2, including accessories and installation elements necessary for stable operation. The accompanying accessories include a power cable that supplies electricity to the system, a load connection cable that links to the electronic load, and banana jack terminals for precise voltage measurement and secure connections. Additionally, a USB cable is provided to connect the testing device to a computer, enabling communication and control via software. The electronic load testing device serves as a simulated load, allowing performance assessment and stability verification during operation. These components must be properly installed before the testing device is put into use.

### 5.2 System description and product overview

**Fig. 3:**
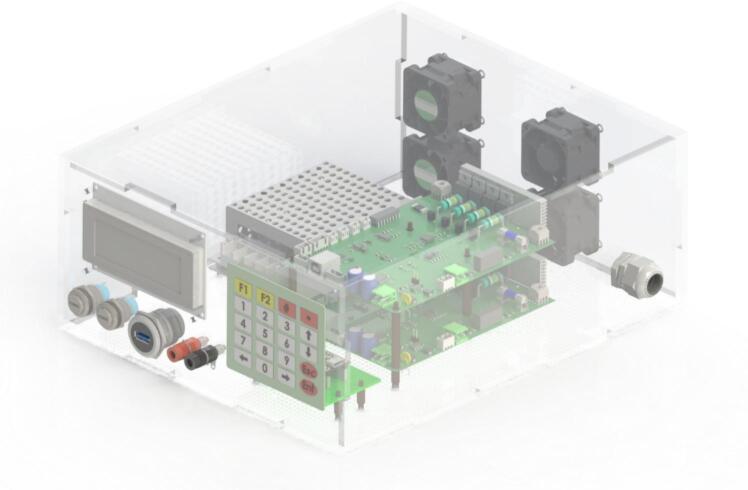
Internal structure of the Electronic Load system with two slave modules.

**Fig. 4:**
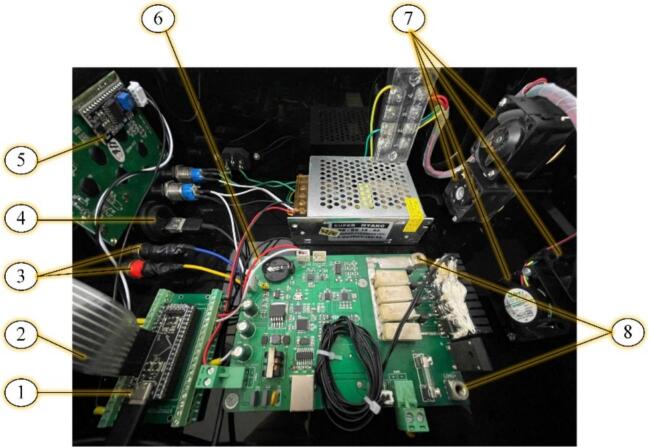
Actual structure of Electronic Load System.

The proposed testing device, illustrated in Fig. 3 and Fig. 4, is designed as an electronic device with a robust protective enclosure that shields internal components from external environmental influences. The front panel features a numeric keypad with function keys (2) for command input, along with a display screen (5) that provides system status and operational information. Additionally, the device is equipped with various connection ports for voltage measurement (3), computer interfacing (4), and load terminals (8) to ensure stable connections with the electronic load.

Inside the system, a main circuit board serves as the central controller (1), responsible for signal processing and overall device operation. An integrated electronic load simulates real-world operating conditions (6), enabling system performance evaluation. Furthermore, the testing device includes a cooling fan (7) to maintain stable operating temperatures, ensuring continuous operation without the risk of overheating.

### 5.3 Installation guide

The testing device is assembled in a well-defined sequence to ensure accuracy and stability during operation. The installation process consists of multiple stages, including assembling the enclosure, securing internal components, and finalizing the connection ports.

**Fig. 5:**
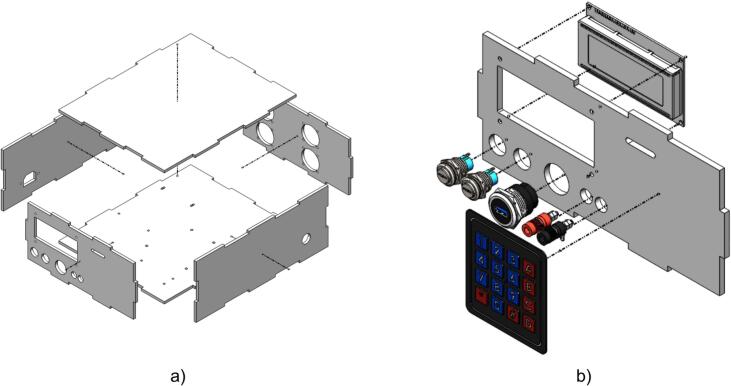
Device assembly: a) Acrylic housing structure; b) Front panel and components.

First, the main enclosure is assembled from panel components designed with interlocking joints, as shown in Fig. 5a. These panels are bonded with adhesive to enhance mechanical durability and ensure structural stability during operation. The assembly process must be carried out carefully, ensuring precise alignment of the joints to maintain the overall integrity of the system.

Once the enclosure is completed, Fig. 5b illustrates the installation of the front panel, which includes control and interface components. Key elements include the display screen, input keypad, banana jack terminals for voltage measurement, power switch, USB port, and control buttons. Each component is positioned in its designated location on the front panel and securely fastened with screws or locking mechanisms to ensure stable operation and ease of use.

**Fig. 6:**
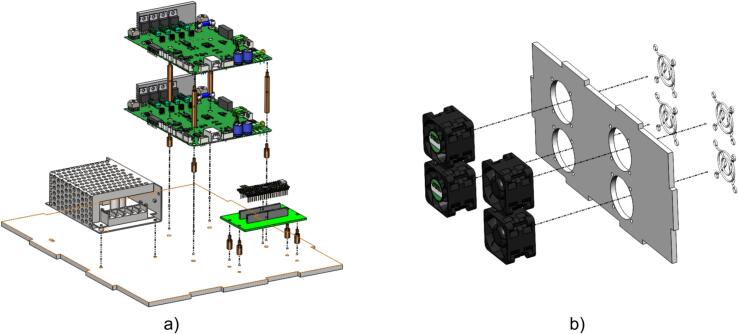
Device assembly: a) Bottom panel and internal components; b) Back panel and cooling fans.

Next, Fig. 6a illustrates the installation of the control circuit board and power supply onto the base panel of the enclosure using brass standoffs. These standoffs create the necessary spacing between components, ensuring sufficient room for wiring and assembly. During installation, the fastening screws must be tightened securely to maintain firm connections and prevent any looseness that could affect system performance.

The cooling fan is mounted on the back of the system to maintain a stable operating temperature. These fans generate airflow to dissipate heat and cool the internal components effectively. During installation, the fan must be firmly secured with screws, and the airflow direction should be checked to ensure optimal cooling efficiency, as shown in Fig. 6b.

**Fig. 7:**
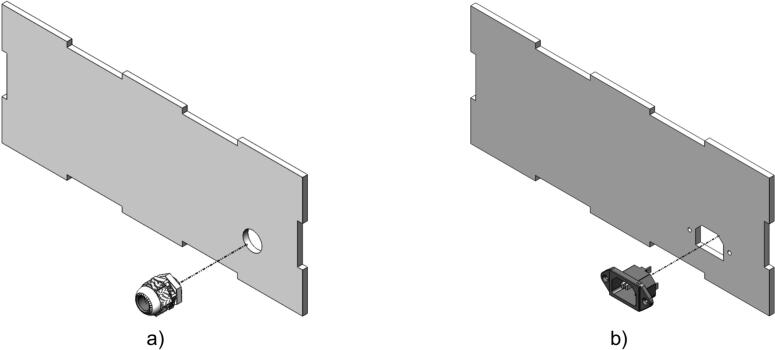
Device assembly: a) Right panel; b) Left panel.

Additionally, the testing device includes connection ports for the discharge load cable and the AC power input, as shown in Fig. 7, which are mounted on the side panel of the device. The discharge load connection ensures stable integration with the electronic load during operation, facilitating performance testing and evaluation. Meanwhile, the AC power input serves as the primary power source for the device and must be securely installed to guarantee both safety and a stable power supply. Both ports are firmly fixed to the side panel, ensuring mechanical durability and convenient connectivity during use.

Upon completing the installation process, a thorough inspection of the entire testing device is required. This includes verifying the secure fastening of all components, checking electrical and mechanical connections, and ensuring overall accuracy and safety before putting the testing device into operation.

### 5.4 System connections

**Fig. 8:**
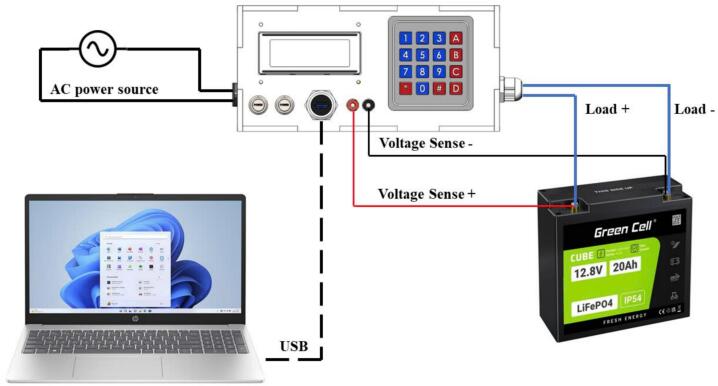
System connection setup.

Before operation, the testing device must be properly connected to all necessary components to ensure accurate functionality. The connections, illustrated in Fig. 8, include power supply setup, computer interface for programming and monitoring, and the configuration of load and voltage measurement cables.

First, the AC power source is connected to the testing device through the dedicated power input located on the side panel of the device. It is essential to ensure that the supplied voltage meets the technical specifications to prevent damage to the components. Once powered, the testing device can be connected to a computer via a USB cable through the communication port, enabling firmware programming, control, and real-time monitoring using specialized software.

Next, the load cable is connected to the discharge load port on the side panel of the testing device. This connection plays a crucial role in evaluating the system's load-handling capabilities. Simultaneously, the voltage measurement cable is attached to the banana jack terminal on the front panel, allowing precise monitoring of the battery voltage during operation. These connections must be securely established to ensure uninterrupted signal transmission and stable system performance.

### 5.5 Internal wiring and component integration

**Fig. 9:**
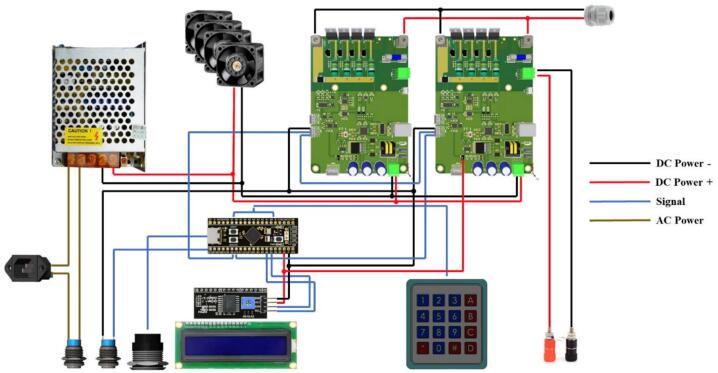
MSEL Wiring Diagram.

Fig. 9 illustrates the internal wiring of the MSEL system, showing the interconnections between the power supply, microcontroller, load modules, cooling fans, and interface components. The power supply converts AC to DC and distributes power to all components, while the microcontroller processes user inputs from the keypad and controls the load modules, displaying system status on the LCD.

### 5.6 Firmware upload guide

**Fig. 10:**
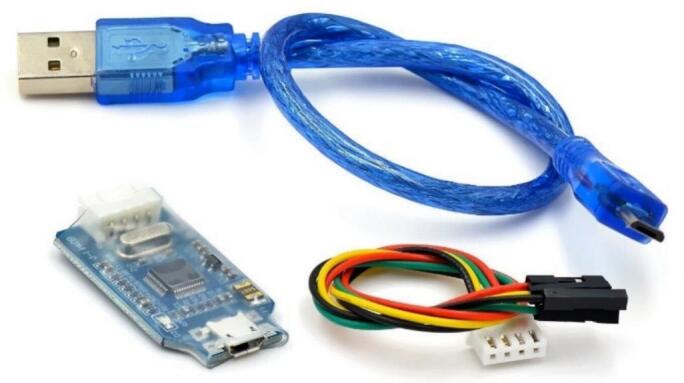
Firmware Upload Accessories: USB-to-SWD/JTAG Debugger and Connection Cables for STM32 Programming.

The process of flashing firmware onto the STM32 microcontroller in the slave circuit and master module via the J-Link interface requires precise connections and a structured procedure. First, all necessary hardware components must be prepared, including slave and master circuits, a J-Link Debugger, and an SWD or JTAG connection cable, as illustrated in Fig. 10. Additionally, a stable power source and a computer are required to manage the programming process. On the software side, SEGGER J-Link Software & J-Flash is used for communication with J-Link, STM32CubeProgrammer for firmware flashing, and Keil MDK, STM32CubeIDE, or IAR Embedded Workbench for compiling the firmware before uploading. Once J-Link is connected to STM32 via the SWDIO, SWCLK, GND, VCC, and NRST pins, the system must be powered on with a stable voltage supply. The connection is verified using J-Link Commander, followed by launching STM32CubeProgrammer or J-Flash, selecting J-Link as the interface, loading the precompiled firmware file, setting the flashing address (typically 0x08000000), and proceeding with the flashing process.

After flashing, the testing device will display a confirmation message, and the verified command can be used to check the firmware integrity. If monitoring is required, J-Link RTT Viewer or UART communication can be utilized. Finally, the J-Link connection is disconnected, and the testing device is restarted to verify proper execution of the program. If errors occur, the wiring, firmware version, or memory status should be checked, and in some cases, the Flash memory may need to be erased before reprogramming. Following this structured approach ensures precise firmware deployment, enabling stable system operation and optimal performance.

## 6 Operation instructions

### 6.1 System state logic and user interaction

**Fig. 11:**
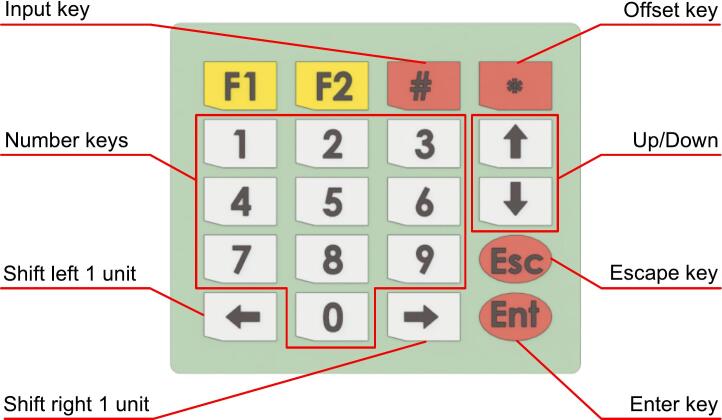
Function mapping of soft matrix 4x5 keypad.

Initially, when the device is powered on, the screen will display a list of five operating modes, with Constant Current mode selected by default. The user can change the mode by pressing a number key from 1 to 5, corresponding to the following modes: Constant Current, Constant Resistance, Constant Power, Battery Capacity, and Battery HPPC. Press Enter to confirm the desired mode. During operating, the testing device provides data streaming via USB. Users can connect the device to a computer and use Data Streamer to record measurement data. Fig. 11 illustrates the keypad layout including function keys for mode selection and data input.

In Constant Current mode, the total current, voltage, and power values are displayed in the screen for monitoring. The user can press '*' on the keypad to offset the reference values of these parameters. To enter a desired load value, press '#', and the testing device will allow data input. The left and right keys can be used to navigate between digits for adjustment. After entering the value, the user can press Enter to save it, or Escape to revert to the previous setting. Once the setup is complete, pressing the Load button will start the operation, and the screen will change from “Load Off” to “Load On”, indicating the active state.

The Constant Resistance and Constant Power modes operate in a similar manner, except that CR mode monitors resistance instead of power.

In Battery Capacity mode, the user must enter the cut-off voltage and maximum current to ensure safe battery testing. Using the up, down, and ‘#’ keys, the user can navigate between parameters to adjust the desired values. These parameters define the operating limits of the battery, preventing undervoltage and excessive current draw. For example, when testing a 2200mAh battery with a 4C peak rating and a safe operating range between 3.7V and 4.2V, the cut-off voltage and maximum allowable current would be 3.7V and 8.8A, respectively. Once the user enters these values and presses Enter, the screen will display the load parameters and user can operate similar to CC mode. Once the battery reaches the cut-off voltage, the testing device will automatically stop. Additionally, the battery capacity value will be continuously updated and displayed on the screen.

Battery HPPC mode is more complex and requires the user to input cut-off voltage, actual battery capacity, rest time between cycles, discharge percentage per cycle, and a selection between 0.5C or 1C discharge rate. Once these values are entered, the testing device automatically calculates the required discharge current and operates according to the Hybrid Pulse Power Characterization (HPPC) method, performing discharge cycles interleaved with rest periods while monitoring battery behavior until reaching the cut-off voltage.

Note: After completing an operation, always turn off the load process before disconnecting the testing device’s power supply. If the Load button is pressed before startup, the testing device will display a warning message, requiring the user to release the button before operation can proceed.

**Fig. 12:**
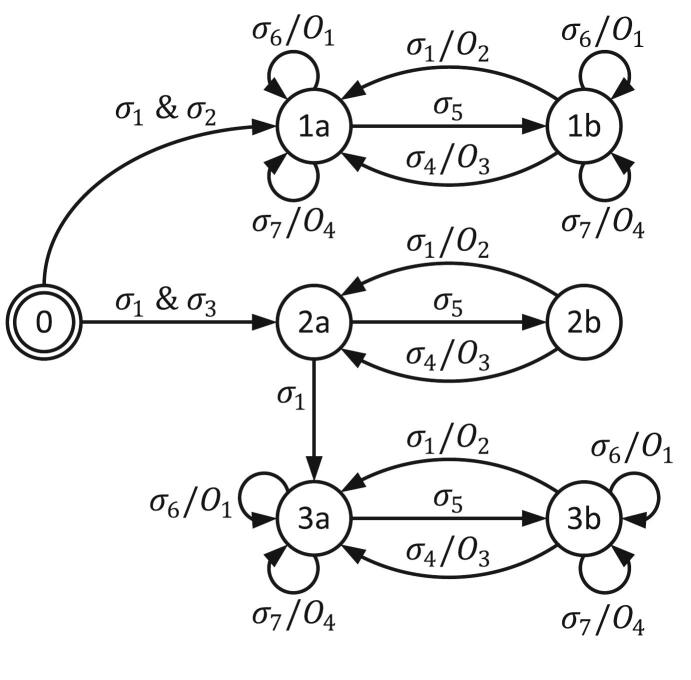
FSM of the MSEL.

The entire operation process follows the Finite State Machine (FSM) shown in Fig. 12, where state 0 is the mode selection state. In state 1a, the system displays real-time measurements from the slave modules and allows the user to operate the load for CC, CR, and CP modes. The user can transition to state 1b to adjust the desired load value. State 2a displays a list of predefined parameters for BC and HPPC modes, with parameter configuration performed in state 2b. For battery-related operation, state 3a provides real-time monitoring and load control, while state 3b is used specifically to modify the target current in BC mode. The inputs and outputs of the FSM are explained in Table 2..

**Table 2:** Symbolic representation of inputs and outputs for the FSM.

Input Symbol	Input Description	Output Symbol	Output Description
$\sigma_{1}$	Enter key pressed	$O_{1}$	Start or stop load operation
$\sigma_{2}$	Mode 1, 2, 3 selected	$O_{2}$	Save parameter input
$\sigma_{3}$	Mode 4, 5 selected	$O_{3}$	Display previous value
$\sigma_{4}$	Escape key pressed	$O_{4}$	Offset voltage and current
$\sigma_{5}$	# key pressed	N/A	-
$\sigma_{6}$	Run button pressed	N/A	-
$\sigma_{7}$	* key pressed	N/A	-

### 6.2 Embedded control loop of slave modules

The primary function of the slave modules is to measure system parameters and regulate the discharge current based on commands received from the master unit. Each slave operates autonomously in a continuous control loop, which includes data acquisition from sensors, real-time current and voltage calculation, and DAC output to control the load. This process ensures accurate current regulation while maintaining responsiveness to commands such as start, stop, or offset initiated by the master.

**Fig. 13:**
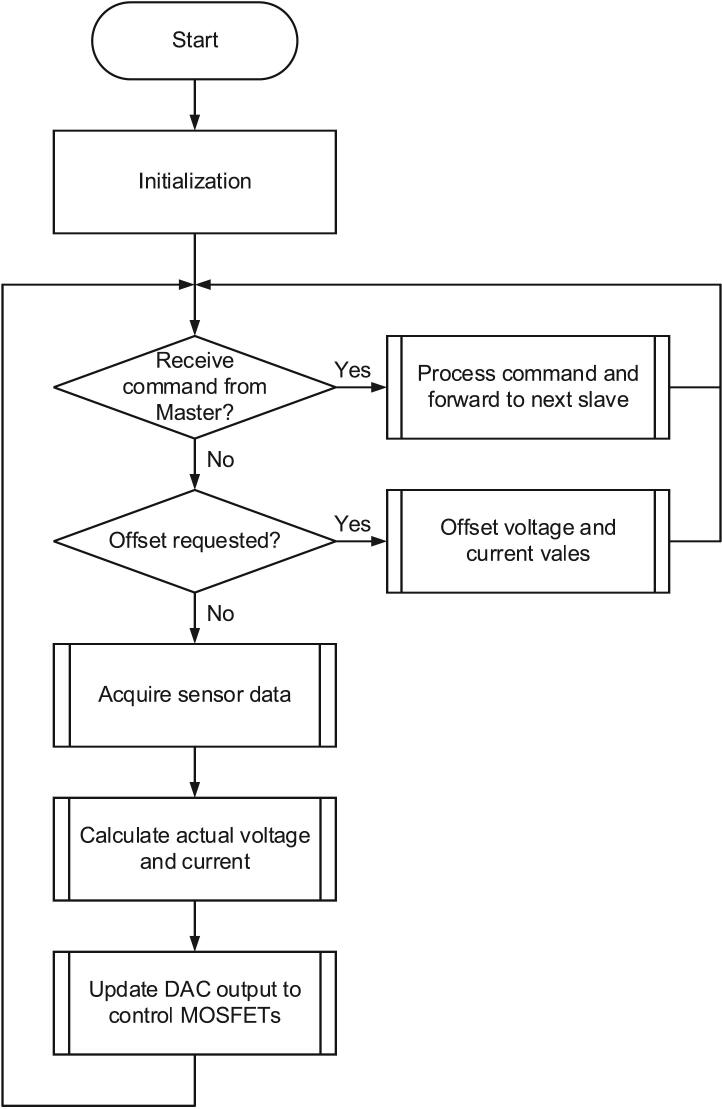
Flowchart of the slave module.

As illustrated in Fig. 13, after system initialization, the slave continuously monitors for incoming commands from the master. When a command is received, the slave processes the request and forwards the updated data along the communication chain. If an offset is requested, the module adjusts its reference values accordingly. Otherwise, it proceeds to acquire sensor data, calculate actual voltage and current, and update the DAC output to regulate the load.

### 6.3 UART communication protocol

The MSEL system utilizes a UART-based daisy chain communication protocol to coordinate control and data exchange between the master unit and multiple slave modules. This architecture enables parallel load operation, real-time monitoring, and modular scalability with minimal wiring complexity. A single command is sent from the master unit through the entire system to both distribute control signals and retrieve measured parameters from the slave modules. The command structure is as follows:

***<Key>:<Current signals>;<Measured currents>;<Measured temperatures>;<Voltage>***.

Where:

<Key>: R (Run) or S (Stop),

<Current signals>: Desired current setpoints for each slave, separated by commas,

<Measured currents>: Actual measured currents from each slave,

<Measured temperatures>: Measured temperatures from each slave,

<Voltage>: Measured voltage from DUT power system.

Since all slave modules are connected in parallel, the system voltage is measured only once, typically by the first slave. The Stop command maintains continuous monitoring of measured parameters without sending active control signals. It follows the same structure as the Run command, with all <Current signals> set to zero to keep the load deactivated.

This command structure inherently enables synchronization across the system via the ring-based daisy chain protocol. As the packet travels sequentially through the chain, each slave extracts its control segment and adds its measurement data to <Measured currents> and <Measured temperatures>. Once the packet completes the loop and returns to the master, the full set of measurement data from all slaves is collected, ensuring system-wide synchronization without requiring additional wiring or external clock signals.

To enhance communication robustness under high-power operating conditions, the MSEL implements a basic error handling mechanism using timeout and retransmission. While the packet structure does not currently include checksum verification, each slave validates received data using formatted parsing. As a result, any deviation from the expected format results in packet rejection.

### 6.4 Data acquisition and external interface

**Fig. 14:**
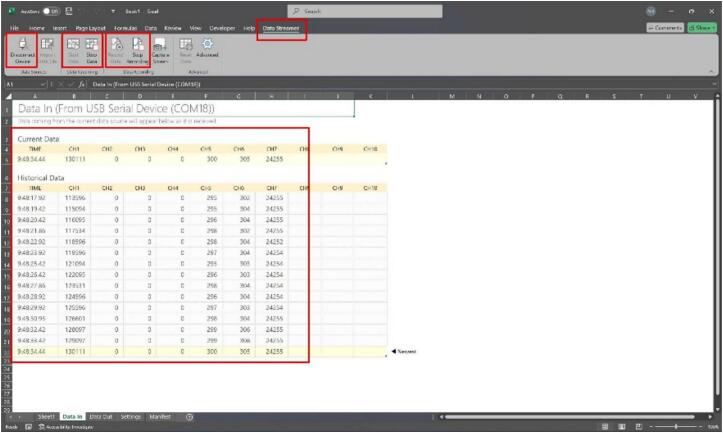
Live measurement data acquisition using Excel Data Streamer interface. Users only need to select the COM port, click Start Data, and the system will automatically transmit real-time parameters for logging and further processing.

The proposed electronic load communicates with external devices via a standard USB connection, presenting itself as a virtual serial port (CDC-ACM). Users can receive real-time measurement data directly through serial monitoring tools, such as terminal emulators or Excel Data Streamer, without requiring dedicated PC software or sending control commands from the host device, as shown in Fig. 14.

Furthermore, the system's text-based data output format allows straightforward integration with external platforms, including Python, LabVIEW, and MATLAB, enabling automated data acquisition and custom testing environments if desired. While no proprietary PC application is provided, the open communication structure ensures broad compatibility for research and development purposes.

## 7 Validation and characterization

### 7.1 Power distribution analysis

The MSEL testing device utilizes multiple slave modules to increase load capacity. However, due to variations in MOSFET internal resistance, wiring losses, and cooling conditions, thermal balance cannot be naturally achieved across all modules. To evaluate the extent of this imbalance, a test was conducted without applying any balancing algorithm. The system operated at a constant 20A load for 30 min using a 3.3V LiFePO4 battery. Along with general system parameters, the current and MOSFET temperature of each module were recorded.

**Fig. 15:**
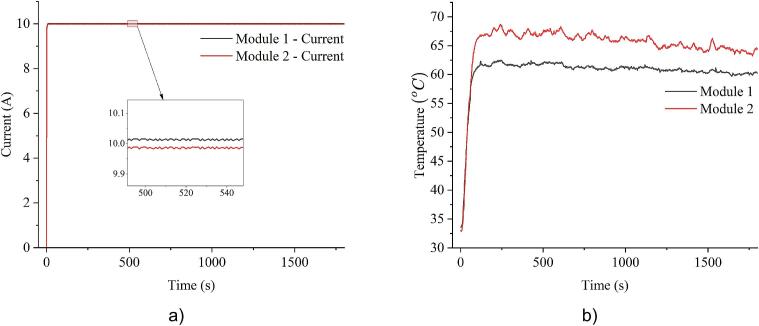
Measured current and temperature of module 1 and 2 under 20A load without balancing algorithm: a) current; b) temperature.

The test results shown in Fig. 15a indicate that while the current was evenly distributed, an imbalance in MOSFET temperature can be seen between the modules. There is not much current imbalance because each module is assigned a desired value algorithmically. However, module 2 operated with a MOSFET temperature approximately $6^{{}^{\circ}}C$ higher than module 1, as illustrated in Fig. 15b. This imbalance comes from physical differences in wiring and cooling condition. Consequently, it could lead to localized overheating, reduced efficiency, and accelerated MOSFET degradation over time.

To address this issue, a dynamic thermal balancing algorithm was implemented to prioritize temperature equalization while keeping current allocation within a reasonable range. The algorithm monitors the current and temperature of each module in real-time and adjusts the load distribution to avoid prolonged stress on any module. Initially, the current is evenly distributed, but once a temperature imbalance is detected, the algorithm starts adjusting the distribution according to the following softmax-based formula (1):

(1) $$\begin{array}{c} P_{i}^{k+1}=P_{i}^{k}+\alpha \left(\frac{e^{-z_{i}}}{\sum_{i}e^{-z_{i}}}P_{total}-P_{i}^{k}\right)\\ z_{i}=w_{I}I_{norm,\;i}+w_{T}T_{norm,\;i} \end{array}$$

In which the normalized current and temperature are defined as follow:

(2) $$\left\{\begin{array}{c} I_{norm,\;i}=\frac{I_{i}}{I_{desired}}\\ T_{norm,\;i}=\frac{T_{i}}{T_{thershold}} \end{array}\right)$$

This approach reduces the power allocation for modules with higher current or temperature. As temperature balancing is prioritized, the temperature weight $w_{T}$ is set higher than the current weight $w_{I}$. This ensures that modules generating more heat are assigned less power in the next iteration.

**Fig. 16:**
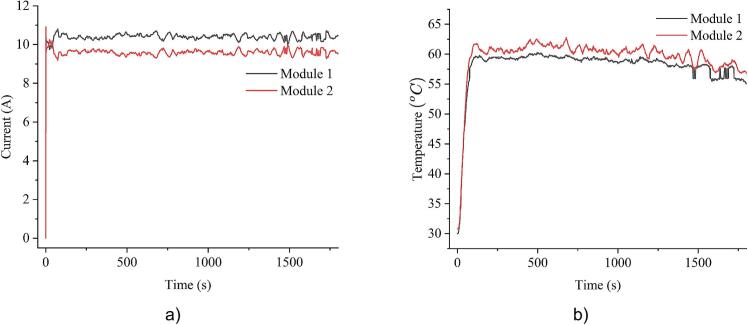
Measured current and temperature of module 1 and 2 under 20A load with balancing algorithm: a) current; b) temperature.

As shown in Fig. 16, the current distribution is adjusted based on the unevenness in temperature. Compared to Fig. 15, the modules operate within a narrower temperature range, with the gap reduced to around $2^{{}^{\circ}}C$. While perfect thermal balance is not achievable due to current allocation constraints, this method clearly improves thermal conditions, helps prevent overheating, and extends the operational lifespan of the MOSFETs.

### 7.2 Application

**Fig. 17:**
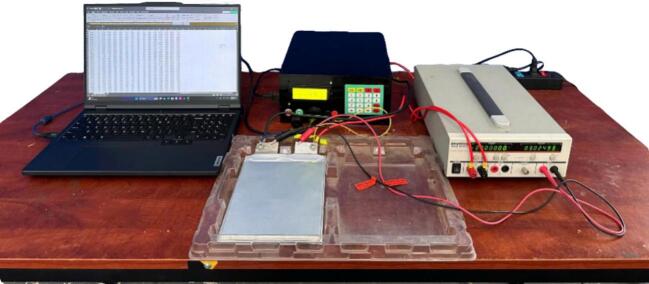
Experimental setup of MSEL with the tested battery, PCS-1000, and a monitoring computer.

The experiment is set up as presented in Fig. 17 to validate the stability, accuracy, and adaptability of MSEL while operating in five different modes under real condition. For CC, CR, and CP modes, the tests were conducted over a 30-minute period to assess the testing device’s ability to maintain load stability. For the battery capacity and HPPC modes, the test was executed until the cut-off voltage of 2.5V was reached to verify the testing device’s functionality in battery applications.

The 123-AMP20M1HD lithium-ion battery, with a nominal voltage of 3.3V and a capacity of 19.5Ah, was used as the test object. During the tests, battery voltage and total current were continuously monitored by the PCS-1000, a high-accuracy meter with current precision of ±0.02% and voltage precision of ±0.005%. The recorded data was used as a reference to validate the control accuracy of the testing device in different modes.

**Fig. 18:**
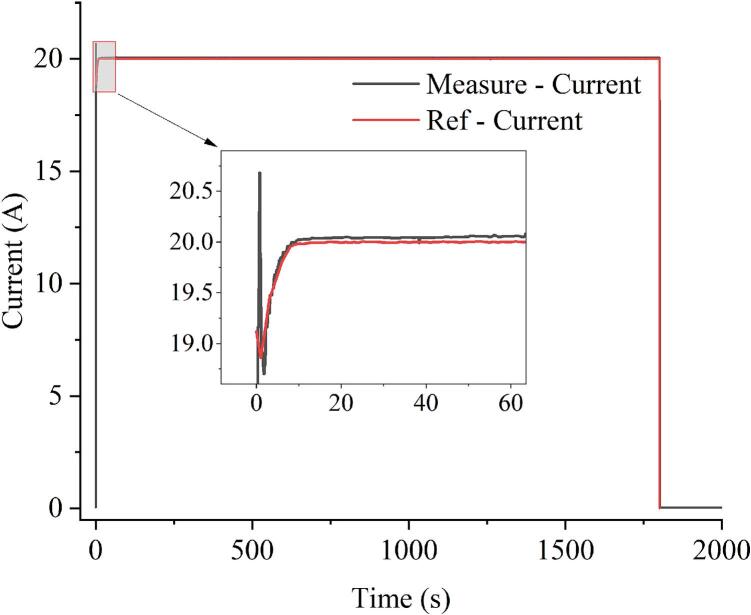
Experimental results of constant current mode at 20A

Fig. 18 presents the results of the Constant Current mode, where the testing device maintained a steady 20A load with fluctuations within ±0.1% of the desired value. This confirms the testing device’s capability to accurately regulate current over extended periods, ensuring load stability even in the variation of voltage and temperature.

**Fig. 19:**
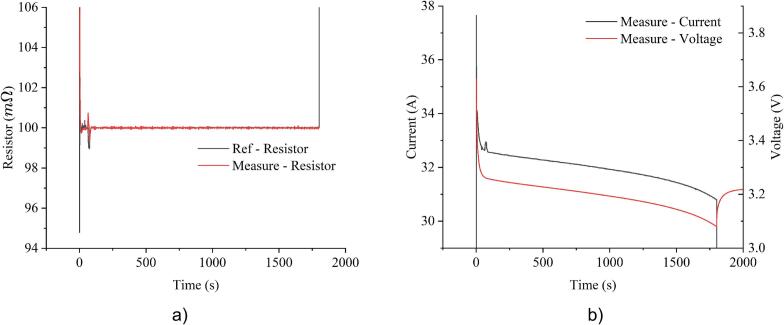
Experimental results of constant resistance mode at 0.1Ω: a) resistance; b) current and voltage.

**Fig. 20:**
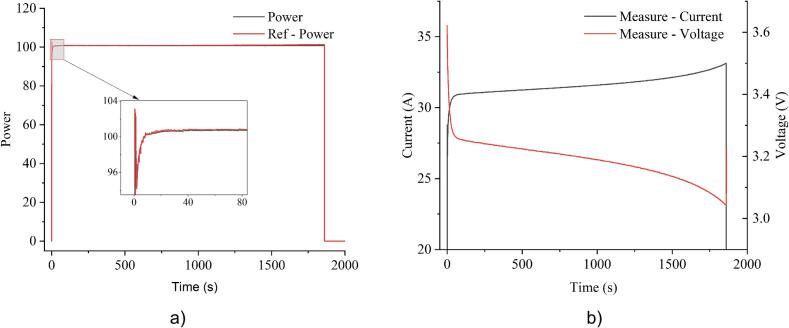
Experimental results of constant power mode at 100W: a) power; b) current and voltage.

In CR mode, the resistance was set to 0.1Ω, resulting in a high starting current at approximately 33A. As the battery voltage gradually decreased, the current correspondingly declined around 31A. Similarly, the variation in rating current is also observed in CP mode, as the system is set to maintain 100W, leading to an increase in current as voltage decline. These behaviors are illustrated in Fig. 19 and Fig. 20. Due to the indirect control through current in CR and CP, their precision was slightly lower than CC mode, with variations of ±0.5%. However, the system maintains overall stable performance, demonstrating its ability to handle different load control strategies.

**Fig. 21:**
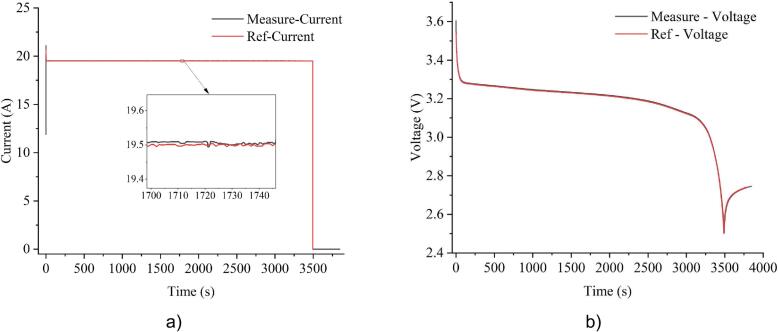
Experimental results of battery capacity mode: a) current; b) voltage.

In regard with the battery-related test, the testing device was evaluated in a real battery discharge scenario. The battery capacity mode was executed by discharging the cell at 1C rate until it reached the cut-off voltage of 2.5V, as shown in Fig. 21. Since this mode relies on integrating discharge current over time, maintaining high measurement accuracy is essential to avoid drift in capacity estimation. The final estimation of battery capacity was 18.918Ah from the system and 18.928Ah from the PCS-1000, resulting in a deviation of ±0.1%. This confirms the system’s ability to perform accurate capacity measurements in real-world battery tests.

**Fig. 22:**
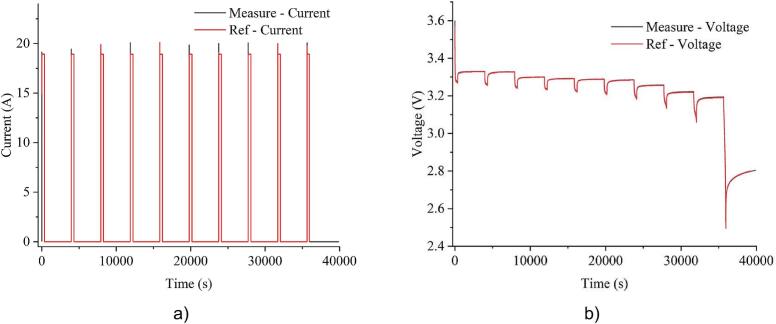
Experimental results of HPPC test mode: a) current; b) voltage.

Fig. 22 illustrates the HPPC test results. In this mode, the testing device automatically calculates the discharge pulses and rest intervals based on preset parameters, including discharge current, rest duration, and percentage of discharge capacity per cycle. The results demonstrate that the current pulses and rest periods are correctly generated, ensuring a structured discharge pattern suitable for battery characterization.

These tests validate that the system operates reliably in battery testing conditions, offering stable current regulation and automated cycle execution, making it applicable for both capacity estimation and dynamic discharge testing.

**Fig. 23:**
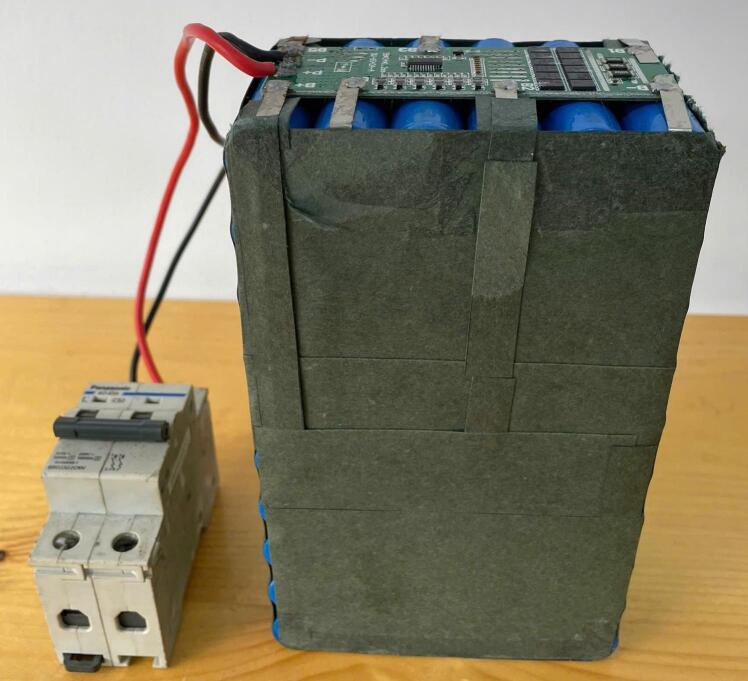
6S10P battery pack consisting of 60 cylindrical 18,650 Li-ion cells.

**Fig. 24:**
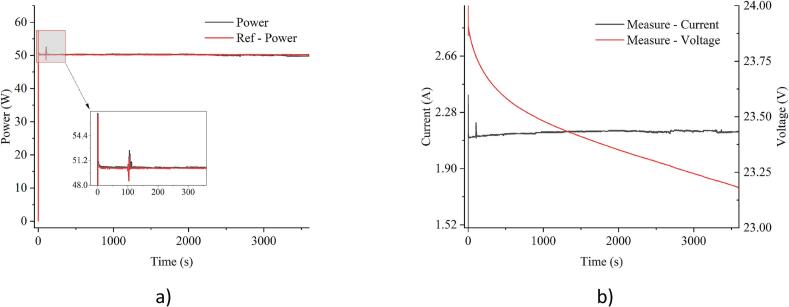
Experimental results of constant power mode at 50W using the 6S10P Li-ion battery pack: a) power; b) current and voltage.

Additionally, a test was conducted using the 6S10P battery pack with a nominal voltage of 22.2V and a maximum of 25.2V, as shown in Fig. 23. This test aimed to verify the system’s compatibility with different battery chemistries and pack configurations beyond a single LiFePO4 battery. During this experiment, the system was set to 50W in CP mode to assess control accuracy under higher voltage conditions. The results in Fig. 24 confirm stable control performance, with both power and voltage regulated effectively throughout the discharge process.

### 7.3 Conclusion

This study presents the design of a cost-effective and scalable electronic load testing device, intended for testing power supplies and batteries. In addition to three standard operating modes commonly found in commercial products, the device provides two specialized modes for battery capacity estimation and model parameter identification. Experimental validation demonstrates an accuracy of ±0.1% in current regulation, ±0.5% in resistance and power control, and ±0.1% in total battery capacity estimation.

The device not only offer various operating modes, but also the ability to increase the power-handle capability. This is achieved by utilizing daisy chain architecture, multiple slave modules can operate together, increasing the total supported load. Furthermore, as an open-source and modular platform, users can customize the testing device easily by modifying only the master unit, rather than the entire system.

Although the testing device has demonstrated stable operation across multiple modes, several areas for future improvement remain. First, its power-handling capability is relatively lower than commercial products of similar size. While scalability allows for higher load capacity, this also increases the overall system size. Another improvement is offering more operating modes, especially complicated and dangerous testing scenario, such as short circuit or overload tests. Finally, the utilization of touchscreen interface instead of a LCD would significantly enhance user experience.

## CRediT authorship contribution statement

**Minh Nhat Huynh:** Visualization, Validation, Software, Investigation, Conceptualization. **Quoc Minh Lam:** Writing – review & editing, Resources. **Cong Toai Truong:** Validation, Data curation. **Huy Hung Nguyen:** Supervision, Formal analysis. **Van Tu Duong:** Writing – review & editing, Validation, Funding acquisition.

## Declaration of competing interest

The authors declare that they have no known competing financial interests or personal relationships that could have appeared to influence the work reported in this paper.
